# Extended Photoperiod Facilitated the Restoration of the Expression of GH-IGF Axis Genes in Submerged Rainbow Trout (*Oncorhynchus mykiss*)

**DOI:** 10.3390/ijms252413583

**Published:** 2024-12-19

**Authors:** Kang Dong, Zhishuai Hou, Zhao Li, Yuling Xu, Qinfeng Gao

**Affiliations:** 1 Key Laboratory of Mariculture, Ministry of Education, Ocean University of China, Qingdao 266003, China; dongkang@stu.ouc.edu.cn (K.D.); zhao-li@stu.ouc.edu.cn (Z.L.); xuyuling@stu.ouc.edu.cn (Y.X.); 2 Function Laboratory for Marine Fisheries Science and Food Production Processes, Qingdao National Laboratory for Marine Science and Technology, Qingdao 266100, China

**Keywords:** submerged rainbow trout, growth, GH-IGF axis, photoperiod

## Abstract

Salmonids, classified as physostomous fish, maintain buoyancy by ingesting air to inflate their swim bladders. Long-term submergence has been shown to cause body imbalance and reduced growth performance in these fish. Previous studies have demonstrated that extended photoperiod can promote growth in salmonids. This study aimed to investigate the regulatory effects of prolonged lighting on the growth of submerged rainbow trout (*Oncorhynchus mykiss*) by examining the transcriptional expression of genes in the growth hormone (GH)-insulin-like growth factor (IGF) axis. Rainbow trout were individually reared in one of the six environments, defined by the combination of three photoperiods (0L:24D, 12L:12D, and 24L:0D) and two spatial rearing modes (routine and submerged), for 16 weeks. We compared the growth performance of rainbow trout in different environments and further analyzed the transcription profiles and correlations of GH-IGF axis genes in the brain, liver, and muscle. The findings of this study were as follows: growth performance of rainbow trout gradually increased with photoperiod duration. Specifically, final body weight (FBW) and specific growth rate (SGR) increased, while feed conversion ratio (FCR) decreased. Extended photoperiod partially mitigated the adverse effects of long-term submergence on rainbow trout growth. Under 24L:0D photoperiod conditions, growth performance (FBW, SGR, and FCR) in submerged and routine rainbow trout was more closely aligned compared to 0L:24D and 12L:12D photoperiod conditions. In response to variations in the photoperiod, GH-IGF axis genes of rainbow trout exhibited significant transcriptional differences, particularly between treatments with 0L:24D and 24L:0D light exposure. An extended photoperiod facilitated the restoration of the expression of GH-IGF axis genes in submerged rainbow trout towards routine levels, including the up-regulation of *sst* and *sstr2* genes in the brain. Correlation analysis implied differentiation of physiological functions of *ghr* and *igfbp* paralogs. This study provided insights into the feasibility of enhancing the growth performance of submerged salmonids through photoperiod manipulation.

## 1. Introduction

Salmonids, comprising species such as salmon, trout, charr, and huchen [1], are considered an excellent source of essential nutrients, including proteins and lipids. Consuming just one or two servings of Atlantic salmon (*Salmo salar*) is suggested to meet an individual’s weekly requirement for eicosapentaenoic acid (EPA) and docosahexaenoic acid (DHA) [2,3,4]. A previous review highlighted the significant roles of EPA and DHA in cardiovascular health, diabetes management, cancer prevention, Alzheimer’s disease, dementia, depression, and in supporting visual and neurological development, as well as maternal and child health [5]. The chemical composition of market-size Atlantic salmon and rainbow trout (*Oncorhynchus mykiss*) was analyzed, revealing that their fillets contained over 18% crude protein and were abundant in essential amino acids like leucine and lysine [6]. Moreover, salmon proteins have demonstrated anti-inflammatory properties, capable of suppressing the expression of tumor necrosis factor-α (TNF-α) and interleukin-6 (IL-6) [7]. Furthermore, salmonids are known for their unique and dynamic flavor. When fresh Atlantic salmon is chewed, free amino acids and 5′-nucleotides are released into the saliva. In particular, glutamic acid and inosine 5′-monophosphate are responsible for umami perception, while alanine and glycine contribute to the sweetness [8]. Due to their nutritional density and palatable taste, salmonids are highly valued worldwide. In 2020, global production of farmed salmonids reached 3.16 million tons, accounting for 37.9% of total marine and coastal farmed finfish production. The export value of salmonids reached USD 27.6 billion worldwide, constituting 18.4% of the total export value of all aquatic products [9].

Surface-based salmonid production is often hindered by issues such as sea lice infestation and unsuitable temperatures, which negatively impact growth performance, welfare, and production of salmonids [10,11,12,13,14,15,16,17]. Culturing salmonids in submerged environments is considered an effective strategy to mitigate these challenges. For instance, Atlantic salmon in submerged sea cages were observed to have lower sea lice loads, with infection levels decreasing as water depth increased [18,19,20]. Previous studies showed that short-term submergence of Atlantic salmon in commercially scaled sea cages does not adversely affect growth performance or welfare [21,22]. These findings demonstrate that short-term submergence culture offers advantages for salmonid production compared to traditional surface farming. However, salmonids have a physostomous swim bladder, which they fill by gulping air at the surface. Air within the swim bladder of Atlantic salmon has been reported to slowly leak out during submersion, becoming completely depleted after 3 weeks [21,23,24,25]. Consequently, submerged Atlantic salmon adopt an unbalanced ‘head-up, tail-down’ swimming posture to compensate for buoyancy when underwater for extended periods [24,26]. Over time, long-term submerged Atlantic salmon displayed slower growth and significantly compressed tail vertebrae compared to their surface counterparts [24]. A 42-day study on submerged Atlantic salmon found that a continuous lighting regimen increased swimming speeds, improved swimming posture, and prevented spinal deformities [25]. Artificial lighting emerged as a promising tool to mitigate the adverse effects of long-term submergence culture on salmonids.

Numerous studies have demonstrated that photoperiod significantly affects growth performance in salmonids [27,28,29,30,31]. Extended exposure to light has been shown to enhance growth by increasing food intake, promoting somatic growth, improving feed conversion efficiency, and suppressing gonadal development [28,30,32,33]. It has been widely recognized that growth is influenced by a multitude of genes and environmental conditions. The growth hormone (GH)-insulin-like growth factor (IGF) axis, similar to its role in mammals, has been identified as playing a major role in growth regulation in teleost fish [34,35]. Research by Yang, et al. [36] demonstrated the axis’s significant role in enhancing skeletal muscle growth, thereby promoting overall sturgeon growth. Consistently, chum salmon (*O. keta*) fry exhibited accelerated growth in seawater, which was associated with the up-regulated expression of genes in the GH-IGF axis [37]. GH-releasing hormone (GHRH), secreted by the hypothalamus, has been shown to trigger the synthesis and release of GH from the pituitary, whereas somatostatin (SST) inhibits these processes. An analysis of pituitary primary cells from the orange-spotted grouper (*Epinephelus coioides*) through in vitro experiments showed that synthetic grouper GHRH exposure induced a dose-dependent enhancement of both *gh* mRNA and GH protein levels [38]. In contrast, SST has been shown to inhibit the synthesis and release of GH in organ-cultured rainbow trout pituitaries [39]. In coho salmon (*O. kisutch*), a dose-dependent increase in body weight and fork length occurred in response to GH injection [40]. Furthermore, the administration of recombinant GH from chum salmon led to a substantial upregulation of *igf-1* mRNA levels in the liver of yellowtail (*Seriola quinqueradiata*) [41]. IGF-1 has emerged as a potential biomarker for assessing instantaneous growth in teleosts, regulating cellular proliferation and correlating with specific growth rates [35,42]. The availability of IGF to receptors was regulated, extending the half-life of IGF through IGF binding protein (IGFBP) [43,44]. The association of IGF with its receptor (IGFR) activated the MAPK/ERK and PI3K/AKT/TOR signaling pathways, thereby promoting nucleic acid and protein synthesis, reducing protein breakdown, and stimulating cellular proliferation, survival, and differentiation, ultimately enhancing fish growth [35,45].

In this study, we compared the growth performance of rainbow trout in different environments and further analyzed the transcription profiles and correlations of GH-IGF axis genes in the brain, liver, and muscle. The research also emphasized the role of extended photoperiod exposure in regulating the transcription of the GH-IGF axis genes in long-term submerged rainbow trout.

## 2. Results

### 2.1. Comparative Analysis of Growth Parameters of Rainbow Trout in Various Environments

**Figure 1:**
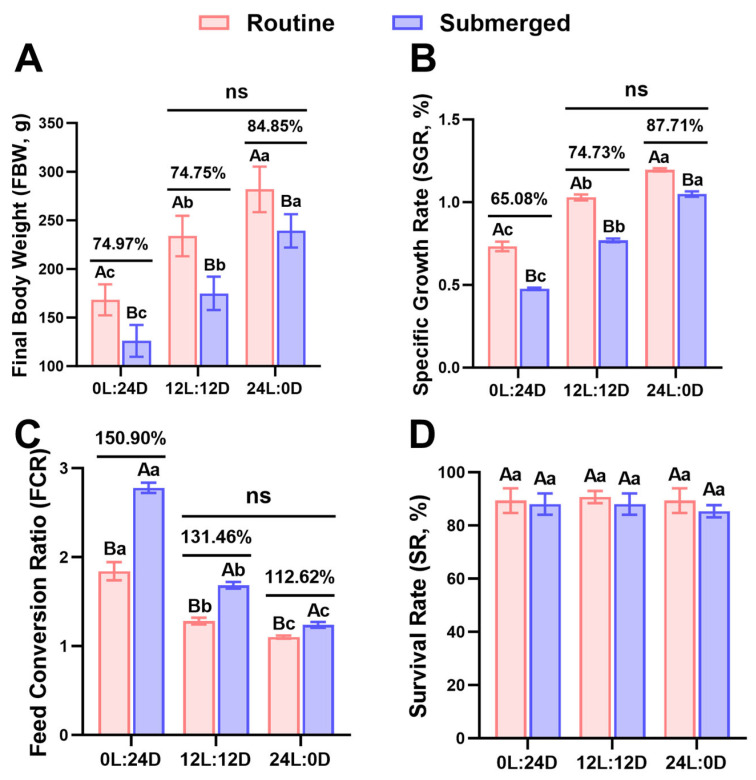
Comparative analysis of growth parameters of rainbow trout in various environments. (**A**) Final body weight (FBW) (*n* = 36). (**B**) Specific growth rate (SGR) (*n* = 3). (**C**) Feed conversion ratio (FCR) (*n* = 3). (**D**) Survival rate (SR) (*n* = 3). The percentages represent the ratios of growth parameters of rainbow trout between submerged and routine modes under the same photoperiod. Data are expressed as means ± standard deviation. Different uppercase letters indicate statistically significant differences between modes under the same photoperiod, while different lowercase letters indicate statistically significant differences among photoperiods under the same mode, *p* < 0.05. “ns” indicates no significant differences between groups.

As shown in Table 1, with the exception of the survival rate (SR), the final body weight (FBW), specific growth rate (SGR), and feed conversion ratio (FCR) of rainbow trout were significantly affected by photoperiod, mode, and their interactive effects (*p* < 0.05). According to Figure 1, the FBW and SGR of rainbow trout showed a significant increase with extended light exposure, whereas the FCR decreased significantly (*p* < 0.05). Compared to the routine group, the FBW and SGR of rainbow trout in the submerged group were significantly lower, whereas the FCR was significantly higher (*p* < 0.05). Furthermore, as light exposure increased, the ratios of FBW and SGR between the submerged and routine groups of rainbow trout gradually increased, while the ratio of FCR decreased progressively. Notably, no significant differences were observed in the FBW, SGR, and FCR between the S24 group and the R12 group of rainbow trout (*p* > 0.05).

### 2.2. Transcriptional Differences of Brain GH-IGF Axis Genes in Various Environments

**Figure 2:**
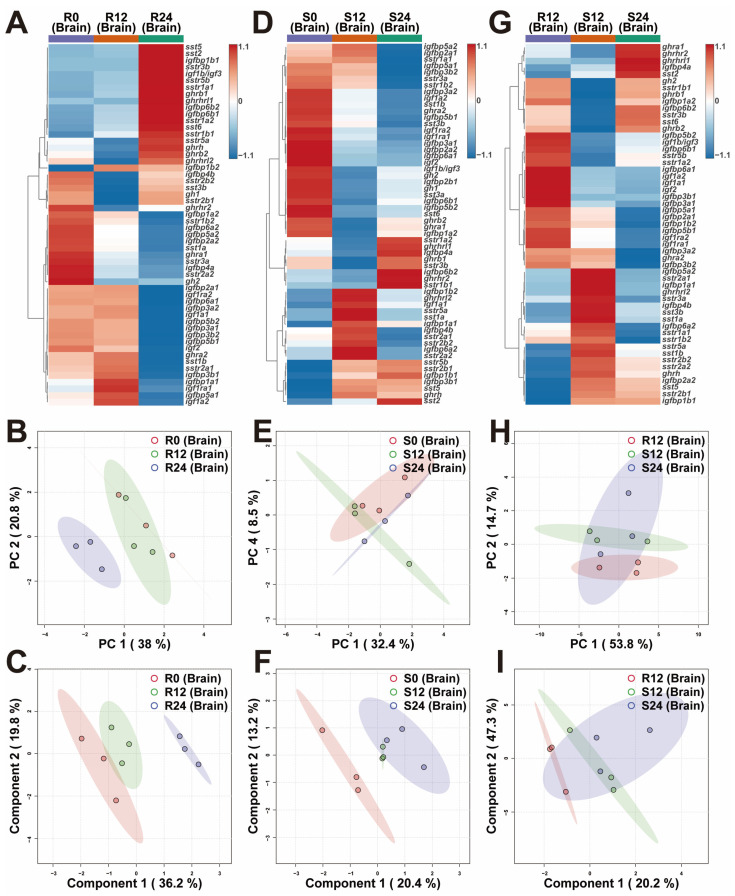
Transcriptional profiles of brain GH-IGF axis genes in various environments. (**A**–**C**) The heatmap (**A**), principal component analysis (PCA) (**B**), and partial least squares discriminant analysis (PLS-DA) (**C**) of the brain GH-IGF axis genes under different photoperiods in routine mode. (**D**–**F**) The heatmap (**D**), PCA (**E**), and PLS-DA (**F**) of the brain GH-IGF axis genes under different photoperiods in submerged mode. (**G**–**I**) The heatmap (**G**), PCA (**H**), and PLS-DA (**I**) of the brain GH-IGF axis genes in interactive environments of photoperiod and mode. R0, 0L:24D photoperiod with routine mode; R12, 12L:12D photoperiod with routine mode; R24, 24L:0D photoperiod with routine mode; S0, 0L:24D photoperiod with submerged mode; S12, 12L:12D photoperiod with submerged mode; S24, 24L:0D photoperiod with submerged mode.

The heatmaps depicted the overall transcriptional differences of brain GH-IGF axis genes in various environments (Figure 2A,D,G). Principal component analysis (PCA) indicated that the R24 group was distinctly separated from both the R0 and R12 groups, while the R0 and R12 groups were closely clustered (Figure 2B). Partial least squares discriminant analysis (PLS-DA) further demonstrated separation among the R0, R12, and R24 groups (Figure 2C). The PCA revealed a separation between the S0 and S24 groups (Figure 2E). The S0 group exhibited clear differentiation from the S12 and S24 groups, with the latter two forming a tight cluster (Figure 2F). Both PCA and PLS-DA revealed a significant distinction between the R12 and S12 groups (Figure 2H,I). Notably, the brains of trout in the S12 group showed up-regulated expression of *sst* (*sst1b* and *sst3b*), *sstr2* (*sstr2a1*, *sstr2a2*, and *sstr2b2*), *sstr3a*, and *sstr5a* genes compared to the R12 group. Conversely, these genes were down-regulated in the S24 group relative to the S12 group (Figure 2G and Figure S1).

### 2.3. Transcriptional Differences of Liver GH-IGF Axis Genes in Various Environments

**Figure 3:**
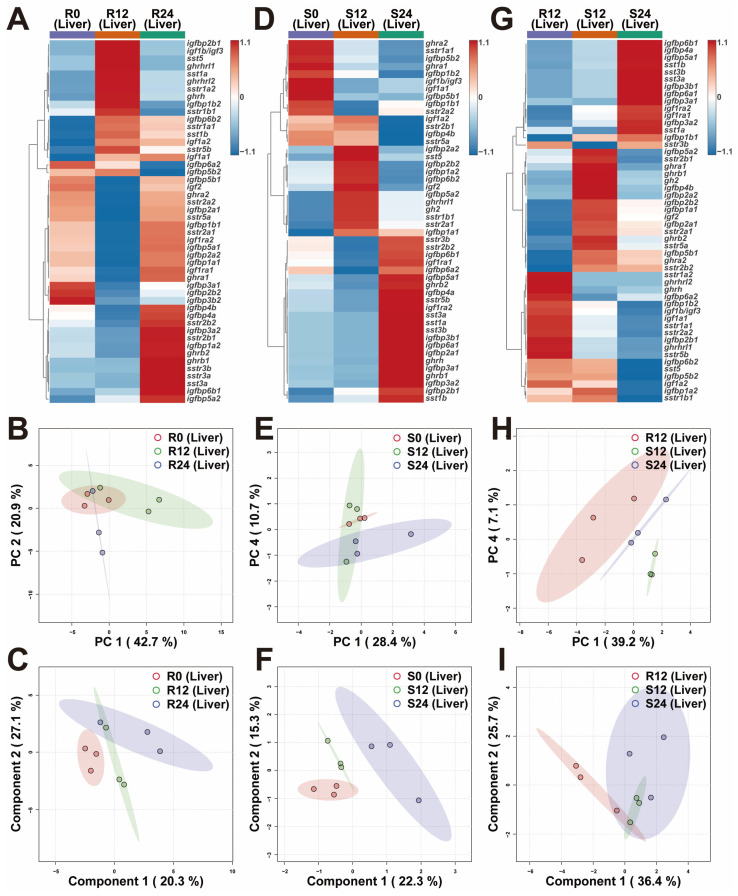
Transcriptional profiles of liver GH-IGF axis genes in various environments. (**A**–**C**) The heatmap (**A**), principal component analysis (PCA) (**B**), and partial least squares discriminant analysis (PLS-DA) (**C**) of liver GH-IGF axis genes under different photoperiods in routine mode. (**D**–**F**) The heatmap (**D**), PCA (**E**), and PLS-DA (**F**) of liver GH-IGF axis genes under different photoperiods in submerged mode. (**G**–**I**) The heatmap (**G**), PCA (**H**), and PLS-DA (**I**) of liver GH-IGF axis genes in interactive environments of photoperiod and mode. R0, 0L:24D photoperiod with routine mode; R12, 12L:12D photoperiod with routine mode; R24, 24L:0D photoperiod with routine mode; S0, 0L:24D photoperiod with submerged mode; S12, 12L:12D photoperiod with submerged mode; S24, 24L:0D photoperiod with submerged mode.

The heatmaps displayed the overall transcriptional differences of liver GH-IGF axis genes in various environments (Figure 3A,D,G). The PCA demonstrated that the R24 group was separated from the R0 and R12 groups (Figure 3B).The PLS-DA confirmed the separation of the R0 group from the R12 and R24 groups, with the R12 and R24 groups clustering together (Figure 3C). The PCA revealed a clear separation between the S0 and S24 groups (Figure 3E). The PLS-DA also demonstrated clear separations among the S0, S12, and S24 groups (Figure 3F). Additionally, PCA highlighted distinct separations among the R12, S12, and S24 groups (Figure 3H). The PLS-DA also demonstrated a separation between the R12 and S12 groups (Figure 3I). Notably, the livers of trout in the S12 group exhibited up-regulation of the *ghra2* gene compared to those in the R12 group. Conversely, the *ghra1* gene was down-regulated in the S24 group relative to the S12 group (Figure 3G and Figure S2).

### 2.4. Transcriptional Differences of Muscle GH-IGF Axis Genes in Various Environments

**Figure 4:**
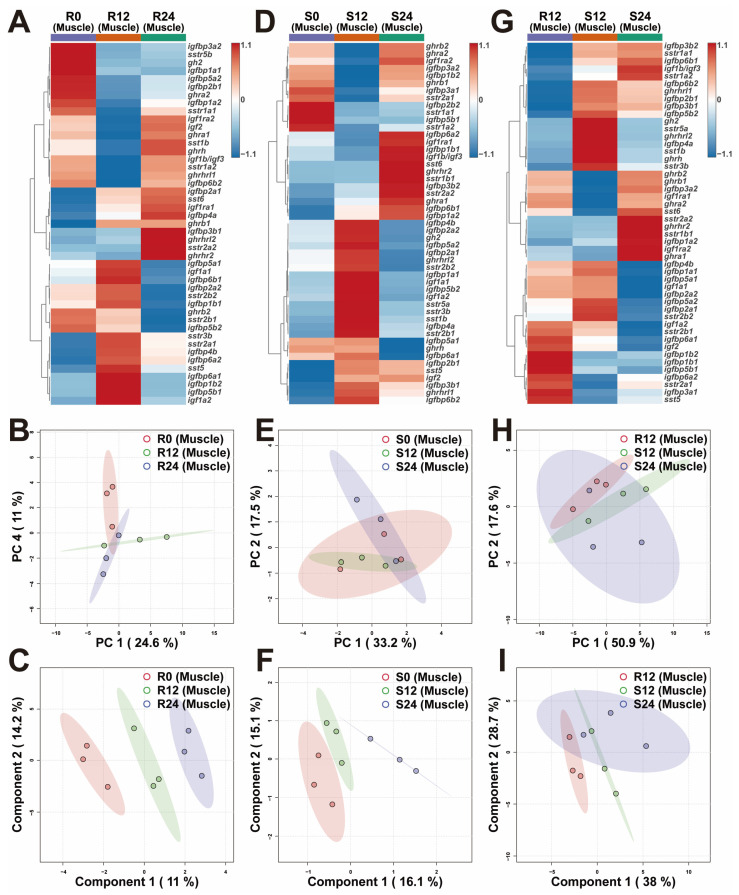
Transcriptional profiles of muscle GH-IGF axis genes in various environments. (**A**–**C**) The heatmap (**A**), principal component analysis (PCA) (**B**), and partial least squares discriminant analysis (PLS-DA) (**C**) of muscle GH-IGF axis genes under different photoperiods in routine mode. (**D**–**F**) The heatmap (**D**), PCA (**E**), and PLS-DA (**F**) of muscle GH-IGF axis genes under different photoperiods in submerged mode. (**G**–**I**) The heatmap (**G**), PCA (**H**), and PLS-DA (**I**) of muscle GH-IGF axis genes in interactive environments of photoperiod and mode. R0, 0L:24D photoperiod with routine mode; R12, 12L:12D photoperiod with routine mode; R24, 24L:0D photoperiod with routine mode; S0, 0L:24D photoperiod with submerged mode; S12, 12L:12D photoperiod with submerged mode; S24, 24L:0D photoperiod with submerged mode.

The heatmaps displayed the overall transcriptional differences of muscle GH-IGF axis genes in various environments (Figure 4A,D,G). The PCA demonstrated that the R0 group was distinctly separated from the R12 and R24 groups (Figure 4B). The PLS-DA confirmed clear separations among the R0, R12, and R24 groups (Figure 4C). The PCA demonstrated that the S24 group was separated from the S12 group (Figure 4E). The PLS-DA validated the separations among the S0, S12, and S24 groups (Figure 4F). Both PCA and PLS-DA revealed a distinct separation between the R12 and S12 groups (Figure 4H,I). Notably, the muscles of trout in the S12 group exhibited up-regulation of the *sstr5a* gene compared to those in the R12 group. Conversely, this gene was down-regulated in the S24 group relative to the S12 group (Figure 4G and Figure S3).

### 2.5. Correlation Analysis of GH-IGF Axis Genes

**Figure 5:**
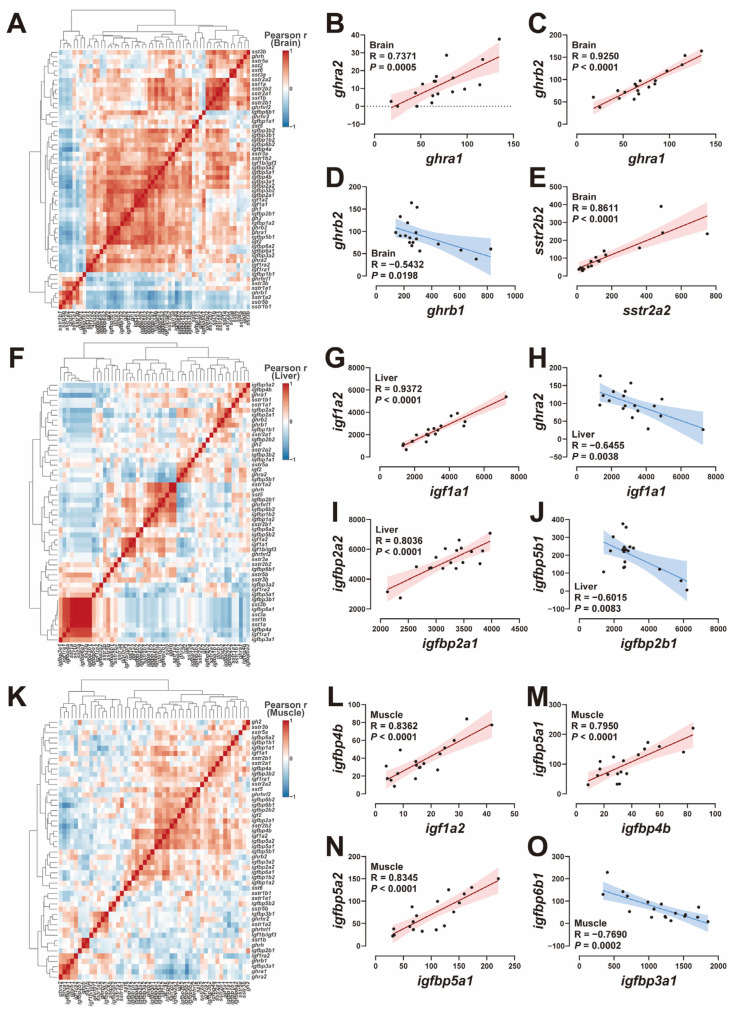
Correlation analysis of GH-IGF axis genes in the brain, liver, and muscle. (**A**–**E**) Heatmap of correlations among GH-IGF axis genes (**A**) and Pearson correlation coefficients of two genes (**B**–**E**) in the brain. (**F**–**J**) Heatmap of correlations among GH-IGF axis genes (**F**) and Pearson correlation coefficients of two genes (**G**–**J**) in the liver. (**K**–**O**) Heatmap of correlations among GH-IGF axis genes (**K**) and Pearson correlation coefficients of two genes (**L**–**O**) in the muscle.

A comprehensive analysis of the transcriptional correlations among GH-IGF axis genes across the brain, liver, and muscle was conducted utilizing hierarchical clustering heatmaps (Figure 5A,F,K). In the brain, *ghra1* exhibited positive correlations with both *ghra2* and *ghrb2* (Figure 5B,C). Conversely, a negative correlation was observed between *ghrb1* and *ghrb2* (Figure 5D). Additionally, *sstr2a2* gene showed a significant positive correlation with *sstr2b2* in the brain (Figure 5E). In the liver, the analysis revealed positive correlations between the expression levels of *igf1a1* and *igf1a2*, as well as *igfbp2a1* and *igfbp2a2* (Figure 5G,I). In contrast, negative correlations were identified between *igf1a1* and *ghra2*, and between *igfbp2b1* and *igfbp5b1* (Figure 5H,J). In muscle tissue, positive correlations were observed between *igf1a2* and *igfbp4b*, *igfbp4b* and *igfbp5a1*, and *igfbp5a1* and *igfbp5a2* (Figure 5L–N). Notably, a negative correlation was identified between *igfbp3a1* and *igfbp6b1* (Figure 5O).

## 3. Discussion

### 3.1. Extended Photoperiod Partially Mitigated the Adverse Effects of Long-Term Submergence on Rainbow Trout Growth

Atlantic salmon raised in short-term submerged cages exhibited growth performance comparable to those reared at the water’s surface, with this approach effectively mitigating the impact of adverse surface conditions [21,22]. However, the growth performance of Atlantic salmon declined with long-term submergence [24], which aligns with the findings of this study. Regardless of photoperiodic conditions, the growth performance of submerged rainbow trout was significantly lower than that of rainbow trout reared under routine conditions (Figure 1). Extended periods of submersion led to Atlantic salmon experiencing negative buoyancy, resulting in a tilted swimming posture, which impaired feeding efficiency and increased energy expenditure due to faster swimming. These factors collectively contributed to reduced growth performance [24]. In this study, we observed that, similar to the impact of photoperiod on the growth performance of routine rainbow trout, the growth performance of submerged rainbow trout also improved with extended light exposure. Furthermore, under long photoperiod conditions, the growth performance of submerged rainbow trout more closely aligned with that of routine rainbow trout compared to short photoperiod conditions. Subsequent analysis revealed that the growth performance of submerged rainbow trout under a 24L:0D photoperiod was equivalent to that of routine rainbow trout under a 12L:12D photoperiod (Figure 1). This interaction suggests that extended light exposure can, to some extent, mitigate the adverse effects of prolonged submergence on rainbow trout growth. Similarly, the 24L:0D photoperiod was reported to reduce the negative impacts of long-term submergence on Atlantic salmon [25].

### 3.2. In Response to the Variations in Photoperiod, the GH-IGF Axis Genes Exhibited Significant Transcriptional Differences

In this study, distinct transcriptional profiles of GH-IGF axis genes were observed in rainbow trout exposed to 0L:24D, 12L:12D, and 24L:0D photoperiods (Figure 2, Figure 3 and Figure 4), indicating a significant response of these genes to photoperiod variations. Similarly, photoperiod was found to influence the circadian patterns of genes and hormones related to the GH-IGF axis in Nile tilapia (*Oreochromis niloticus*) [46]. Fluctuations in plasma GH and IGF levels in adult Atlantic salmon were also reported to correlate with photoperiod changes [47]. Moreover, previous studies have demonstrated that photoperiod plays a crucial role in regulating fish growth performance. A positive effect of long daylight hours on growth was observed in Largemouth bass (*Micropterus salmoides*) [48], juvenile gibel carp (*Carassius auratus*) [49], winter flounder (*Pseudopleuronectes americanus*) [50], dusky grouper (*Epinephelus marginatuswas*) [51], brook trout (*Salvelinus fontinalis*) [52], and zebrafish (*Danio rerio*) [53]. For blunt snout bream (*Megalobrama amblycephala*) [54] and tomato clownfish (*Amphiprion frenatus*) [55], long photoperiod conditions led to higher expression levels of GH-IGF axis genes and improved growth performance. Additionally, the growth-promoting effects of the GH-IGF axis have been confirmed in sturgeon [36]. Based on these findings, we hypothesize that photoperiod regulates rainbow trout growth through the modulation of the GH-IGF axis.

### 3.3. Extended Photoperiod Facilitated the Restoration of the Expression of GH-IGF Axis Genes in Submerged Rainbow Trout Towards Routine Levels

Previous studies have demonstrated that salmonids develop body imbalance and exhibit poor growth when cultured in submerged sea-cages for over a month [24,26]. In the current study, significant differences were identified in the transcriptional profiles of GH-IGF axis genes between trout under R12 and S12 conditions (Figure 2, Figure 3 and Figure 4). Notably, trout in the S12 group showed up-regulated expression of *sst* (*sst1b* and *sst3b*) and *sstr2* (*sstr2a1*, *sstr2a2*, and *sstr2b2*) genes in the brain compared to the R12 group. However, these genes were down-regulated in the S24 group relative to the S12 group (Figure 2 and Figures S1). Prior research showed that the injection of SST-14 into rainbow trout reduced plasma levels of GH and IGF-1, while long-term implantation of SST-14 led to decreased food conversion efficiency and significant growth retardation. These findings suggest that SST inhibits rainbow trout growth by modulating the GH-IGF axis [56]. Supporting this, a study involving *sstr2* gene knockout in piglets demonstrated increased body weight, indicating that reduced SST signaling can improve growth [57]. Conversely, the overexpression of sstr2 in rat pituitary somatotroph tumor cells resulted in decreased *gh* mRNA levels and reduced GH secretion [58]. In rainbow trout hepatocytes, SST diminished GH responsiveness by promoting GHR internalization and downregulating *ghr* transcription [59]. SST binding to its receptor SSTR2 was also found to activate the ERK signaling pathway, further inhibiting *ghr* expression [60]. Stress [61] and long-term fasting [62] resulted in reduced *ghr* expression in salmonids. By contrast, in grass carp (*Ctenopharyngodon idellus*), a higher growth rate was associated with *ghr* upregulation [63]. Interestingly, in this study, the livers of trout in the S12 group exhibited up-regulation of the *ghra2* gene compared to those in the R12 group, whereas *ghra1* gene was down-regulated in the S24 group relative to the S12 group (Figure 3 and Figure S2). These discrepancies with prior studies may reflect the functional divergence among *ghr* gene subtypes in fish. For example, in gilthead sea bream (*Sparus aurata*), opposing expression patterns of *ghr1* and *ghr2* were observed: *ghr1*, which promotes growth, was up-regulated, while *ghr2*, associated with growth inhibition, was down-regulated during development [64]. Similarly, sustained exercise in gilthead sea bream down-regulated *ghr2* expression in muscle, correlating with enhanced growth [65], while crowding stress upregulated *ghr2* in the liver [66,67]. Additionally, fasting was found to increase muscle *ghr2* expression in both rainbow trout [68] and gilthead sea bream [69].

### 3.4. The Correlation of GH-IGF Axis Genes

Salmonids have undergone four rounds of genome duplication, resulting in multiple paralogs of individual genes. Correlation analysis of *ghr* subtypes in the brain suggested that *ghrb1* may have functionally differentiated from *ghra1*, *ghra2*, and *ghrb2* (Figure 5B–D). A similar phenomenon was observed in gilthead sea bream, where *ghr1* promoted growth, while *ghr2* inhibited growth [64]. The negative correlation between *ghra2* and *igf1a1* in the liver (Figure 5H) indicated that *ghra2* might contribute to growth inhibition. This hypothesis was supported by the alterations in hepatic *ghra2* expression following R12, S12, and S24 treatments (Figure 3 and Figure S2). In salmonids, IGFBP-2B is one of the primary carriers of circulating IGF and is produced specifically by the liver [70,71]. The down-regulation of *igfbp2b* in the liver of Atlantic salmon following post-fasting refeeding indicates its growth inhibitory effect [71]. Conversely, the upregulation of *igfbp5* in the liver of grass carp after GH injection indicates a growth-promoting function [72]. These findings align with the negative correlation between *igfbp2b1* and *igfbp5b1* expression observed in the present study (Figure 5J). In muscle, a positive correlation between *igfbp4b* and *igf1a2* was identified (Figure 5L), suggesting a growth-promoting effect. This is consistent with prior studies in Atlantic salmon, where significant positive correlations between *igfbp4* and genes involved in muscle development were observed during in vitro myogenesis [73]. In the musculature of coho salmon, the expression levels of *igfbp3a1* were elevated as a result of GH transgenesis, which is indicative of its potential to stimulate growth [74]. In addition, the expression of *igfbp3a1* and *igfbp6b1* was negatively correlated in this study (Figure 5O). Elevated expression levels of either zebrafish *igfbp6* paralogs led to a notable decrease in embryonic growth, implying a potential role in inhibiting growth [75].

## 4. Materials and Methods

### 4.1. Ethics Statement

The research adhered to the ethical guidelines established by the Animal Research and Ethics Committee at Ocean University of China (permit 2014201). Additionally, all procedures complied with the protocols for laboratory animal care outlined by the National Institutes of Health (NIH), as specified in NIH publication No. 8023, revised in 1987.

### 4.2. Fish Stock and Acclimation

**Figure 6:**
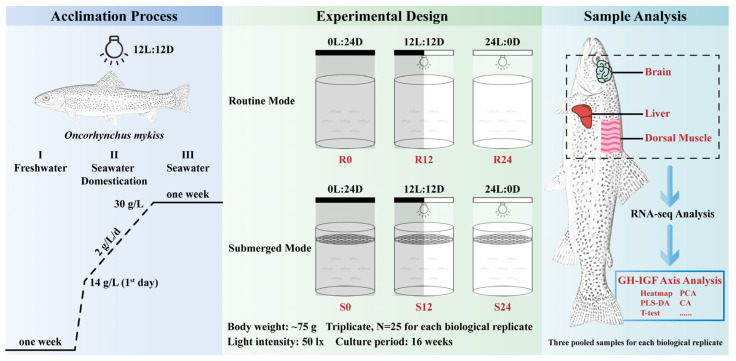
Schematic representation of the experimental procedure. (**Left**) The trout were subjected to a series of acclimation steps prior to the experiment: (I) Freshwater environment. (II) Seawater domestication. (III) Seawater environment. (**Middle**) Experimental design. Six groups were set up with triplicates: 0L:24D photoperiod with routine mode (R0), 12L:12D photoperiod with routine mode (R12), 24L:0D photoperiod with routine mode (R24), 0L:24D photoperiod with submerged mode (S0), 12L:12D photoperiod with submerged mode (S12), and 24L:0D photoperiod with submerged mode (S24), respectively. (**Right**) Brain, liver, and dorsal muscle tissues were dissected for the analysis of the GH-IGF axis genes.

For this study, diploid rainbow trout underyearlings were procured from Linqu Salmon and Trout Farm in Weifang, Shandong, China, and then cultured at Haiyang Yellow Sea Aquatic Product Co., Ltd. in Yantai, Shandong, China. The trout were acclimated to laboratory conditions over three stages (Figure 6):

(I) They were initially reared in a freshwater system for 1 week to acclimatize to a new environment.
(II) Next, the trout underwent a process of seawater domestication, where the freshwater in the rearing system was gradually replaced with deep-well seawater, which had a salinity of approximately 30 g/L. Specifically, on the first day of this process, a substantial amount of seawater was introduced into the rearing system, immediately raising the salinity to 14 g/L. Thereafter, seawater was incrementally added, increasing the rearing water’s salinity by 2 g/L each day, until the rearing system was completely filled with seawater.
(III) Finally, the trout were retained in seawater for a further week to ensure complete adjustment.

During the acclimation phases, the trout were exposed to a light-dark cycle of 12 h light to 12 h darkness (12L:12D) and were provided with a commercial feed, constituting about 1.5% of their body weight, which was administered twice each day at 9:00 a.m. and 3:00 p.m. The water temperature was meticulously regulated to range from 15 to 16 °C, and the concentration of dissolved oxygen was sustained above 7 mg/L.

### 4.3. Experimental Design and Sample Collection

In this study, three photoperiodic treatments were established: continuous darkness (0L:24D), 12L:12D, and continuous 24-h lighting (24L:0D). Additionally, two spatial rearing modes were employed: a routine mode (R) that permitted the trout access to the water surface, and a submerged mode (S) that prevented the trout from reaching the surface. Consequently, the experimental setup included six groups, each with three replicates, characterized by the following conditions: 0L:24D photoperiod with routine mode (R0), 12L:12D photoperiod with routine mode (R12), 24L:0D photoperiod with routine mode (R24), 0L:24D photoperiod with submerged mode (S0), 12L:12D photoperiod with submerged mode (S12), and 24L:0D photoperiod with submerged mode (S24) (Figure 6). Following acclimation, 25 trout averaging around 75 g were randomly assigned to each replicate tank, which had a volume of 500 L and was continuously supplied with deep-well seawater. Illumination was supplied by a 3 W white light-emitting diode (LED) bulb (E27, Bull, Cixi, China) positioned above the water surface and controlled by an electronic timer (GND-1, Bull). The light intensity at the water’s surface was maintained at 50 lux, as ascertained by an illuminance meter (VC1010C, Victor, Shenzhen, China). Trout designated for submerged conditions were enclosed beneath the waterline using a polyethylene mesh with one-centimeter apertures, which efficiently precluded their ability to reach the surface for air intake. The availability of feed, water temperature, and level of dissolved oxygen were maintained in accordance with the conditions established during the acclimation period.

Following the 16-week experimental phase, the trout were subjected to a 24-h fast before being anaesthetized with 3-Aminobenzoic acid ethyl ester methanesulfonate (MS-222). Thereafter, 12 trout per tank were weighed, and three individuals from each tank were randomly selected for the procurement of brain, liver, and dorsal muscle tissues, as illustrated in Figure 6. The harvested tissues were placed in enzyme-free tubes, rapidly frozen in liquid nitrogen, and subsequently stored at −80 °C for RNA-seq analysis.

### 4.4. Calculation of Growth Parameters

The growth parameters were calculated using the following formulas:

SGR (%) = (lnFBW − lnIBW)/t × 100;

FCR = FG/(FBW − IBW);

SR (%) = FN/IN × 100.

where: SGR, specific growth rate; FCR, feed conversion ratio; SR, survival rate; FBW, final body weight; IBW, initial body weight; t, days of culture; FG, feed given; FN, final number of fish; IN, initial number of fish.

### 4.5. RNA-Seq Analysis

RNA isolation from the brain, liver, and muscle was carried out using TRIzol^®^ Reagent in line with the protocol provided by Invitrogen (Carlsbad, CA, USA), with DNase I from TaKara (Tokyo, Japan) employed to eliminate genomic DNA contamination. The RNA’s integrity and concentration were evaluated with a 2100 Bioanalyzer by Agilent (Santa Clara, CA, USA) and an ND-2000 spectrophotometer from NanoDrop Technologies (Wilmington, DE, USA), respectively. For library construction, only RNA samples of exceptional quality were selected, exhibiting an OD260/280 ratio between 1.8 and 2.2, an OD260/230 of at least 2.0, an RNA Integrity Number (RIN) above 6.5, a 28S:18S ratio greater than or equal to 1.0, and a weight exceeding 1 μg. To minimize biological variability, equal volumes of RNA from three individuals within each replicate tank were pooled to form a single RNA-Seq library, which was prepared using Illumina’s TruSeqTM RNA Sample Preparation Kit (San Diego, CA, USA). The library was size-selected for cDNA fragments of 300 bp using a 2% Low Range Ultra Agarose gel (Bio-Rad, Hercules, CA, USA), followed by PCR amplification for 15 cycles with Phusion DNA polymerase from NEB (Beverly, MA, USA). Quantification was performed using a TBS380 instrument (Turner Biosystems, Sunnyvale, CA, USA), and the paired-end library was then sequenced using an Illumina HiSeq xten/NovaSeq 6000 platform with a read length of 2 × 150 bp. Raw sequencing reads were processed for quality trimming and control with SeqPrep (https://github.com/jstjohn/SeqPrep, accessed on 15 January 2023) and Sickle (https://github.com/najoshi/sickle, accessed on 15 January 2023) using default settings. The clean reads were then aligned to the rainbow trout reference genome (GCF_013265735.2) using HISAT2 (Version 2.1.0) in orientation mode [76]. Gene abundances were quantified utilizing the RSEM algorithm (Version 1.3.3) [77].

### 4.6. qPCR Validation of the Transcriptome

Total RNA was extracted from the brain, liver, and muscle using the reagent TRIzol (Takara). Equal amounts of RNA (500 ng) were reverse-transcribed into cDNA using the HiScript III RT SuperMix for qPCR (+gDNA wiper) (R323, Vazyme, Nanjing, China) following the manufacturer’s instruction. The primers of the target genes and reference genes *β-actin* were designed using sequences from rainbow trout reference genome (GCF_013265735.2) with Primer Premier 6.0 software (Premier Biosoft International, Palo Alto, CA, USA, Table S1). Real-time qPCR reactions (20 μL) were performed with ChamQ SYBR Color qPCR Master Mix (Low ROX Premixed) (Q431, Vazyme). Each reaction contained: 10 μL of 2× ChamQ SYBR Color qPCR Master Mix (Low ROX Premixed), 2 μL of cDNA, 0.4 μL of each primer (forward and reverse), and ddH_2_O to adjust the total volume to 20 μL. The thermal profile for qPCR was as follows: 30 s at 95 °C, followed by 40 cycles of 95 °C for 10 s and 60 °C for 30 s. A melt curve analysis was then performed to confirm the amplification of a single product. The relative mRNA expression levels of the target genes were normalized to the geometric mean expression levels of *β-actin* by using the formula 2^−ΔΔCt^. The validation results were displayed in Figure S4.

### 4.7. Statistical Analysis

Statistical analysis of growth data was performed using SPSS 26.0 (IBM, Armonk, NY, USA). Data normality was assessed using the Shapiro–Wilk test. If the data did not meet the criteria for normal distribution, non-parametric tests were utilized. For normally distributed data, analysis of variance (ANOVA) was conducted, and the homogeneity of variances was evaluated using the Levene test. Post-hoc multiple comparisons were performed using Tukey’s HSD equal variances and Tamhane’s T2 unequal variances. A *p*-value less than 0.05 was considered statistically significant. Data are presented as mean ± standard deviation. RNA-Seq data were further analyzed using MetaboAnalyst’s online platform (https://www.metaboanalyst.ca, accessed on 6 June 2024), employing various analytical methods. These included clustering algorithms for heatmap generation, principal component analysis (PCA), partial least squares discriminant analysis (PLS-DA), and correlation analysis (CA). Differential expression analysis was performed using DESeq2 (Version 1.24.0), utilizing a negative binomial distribution model to evaluate expression differences based on read count data aligned with specific genes.

## 5. Conclusions

The growth performance of rainbow trout was gradually enhanced with photoperiod. An extended photoperiod somewhat reduced the adverse effects of long-term submergence on rainbow trout growth. In response to the variations in photoperiod, the GH-IGF axis genes of rainbow trout exhibited significant transcriptional differences, particularly between treatments involving darkness and continuous light exposure. An extended photoperiod facilitated the restoration of the expression of GH-IGF axis genes in submerged rainbow trout towards routine levels. Correlation analysis implied differentiation of physiological functions of *ghr* and *igfbp* paralogs.

## Figures and Tables

**Table 1:** *p*-values from two-way ANOVA: Effects of photoperiod and mode on growth parameters in rainbow trout.

Parameters	FBW (g)	SGR (%)	FCR	SR (%)
Photoperiod	0.000	0.000	0.000	0.656
Mode	0.000	0.000	0.000	0.159
Photoperiod × Mode	0.009	0.000	0.000	0.831

Note: FBW, final body weight; SGR, specific growth rate; FCR, feed conversion ratio; SR, survival rate.

## Data Availability

Data are contained within the article.

## Supplementary Materials

The following supporting information can be downloaded at: https://www.mdpi.com/article/10.3390/ijms252413583/s1.
