# Supplementary material for: Extended Photoperiod Facilitated the Restoration of the Expression of GH-IGF Axis Genes in Submerged Rainbow Trout (Oncorhynchus mykiss)
Source: Int J Mol Sci. 2024 Dec 19;25(24):13583. doi: 10.3390/ijms252413583 (PMC11679508; doi:10.3390/ijms252413583)
Supplement: Supplementary file 1 [file ijms-25-13583-s001.zip › ijms-3368751-supplementary.pdf]

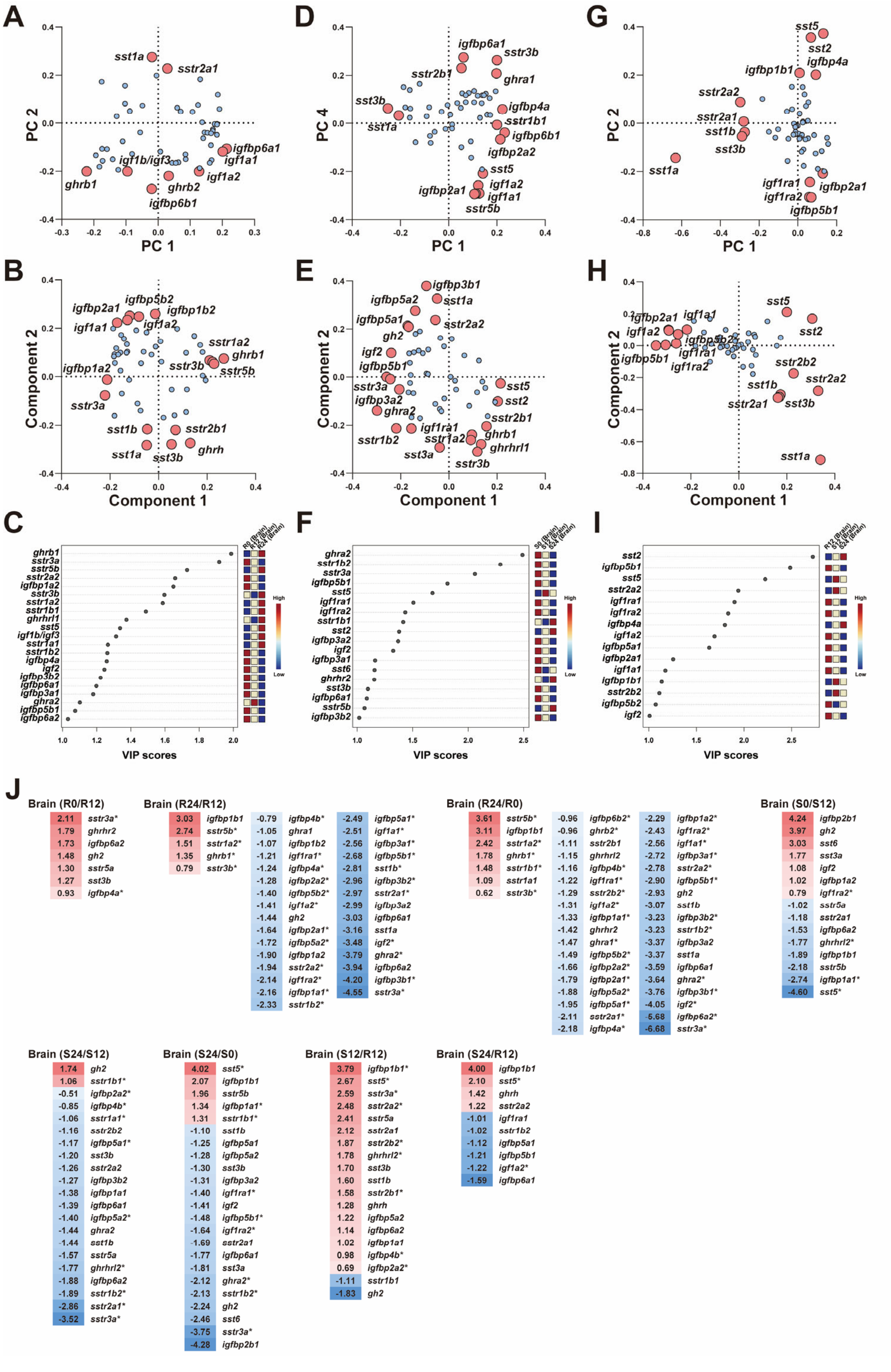

Figure S1. The key genes with changes in the brain GH-IGF axis in various environments. (**A-C**) PCA loading plot (**A**), PLS-DA loading plot (**B**), and variable importance in projection (VIP) values (**C**) of brain GH-IGF axis genes under different photoperiods in routine mode. (**D-F**) PCA loading plot (**D**), PLS-DA loading plot (**E**), and VIP values (**F**) of brain GH-IGF axis genes under different photoperiods in submerged mode. (**G-I**) PCA loading plot (**G**), PLS-DA loading plot (**H**), and VIP values (**I**) of brain GH-IGF axis genes in interactive environments of photoperiod and mode. (**J**) Differential expression between brain GH-IGF axis genes in the two groups. The numbers represent log2(fold change) values, and "*" indicates $P < 0.05$.

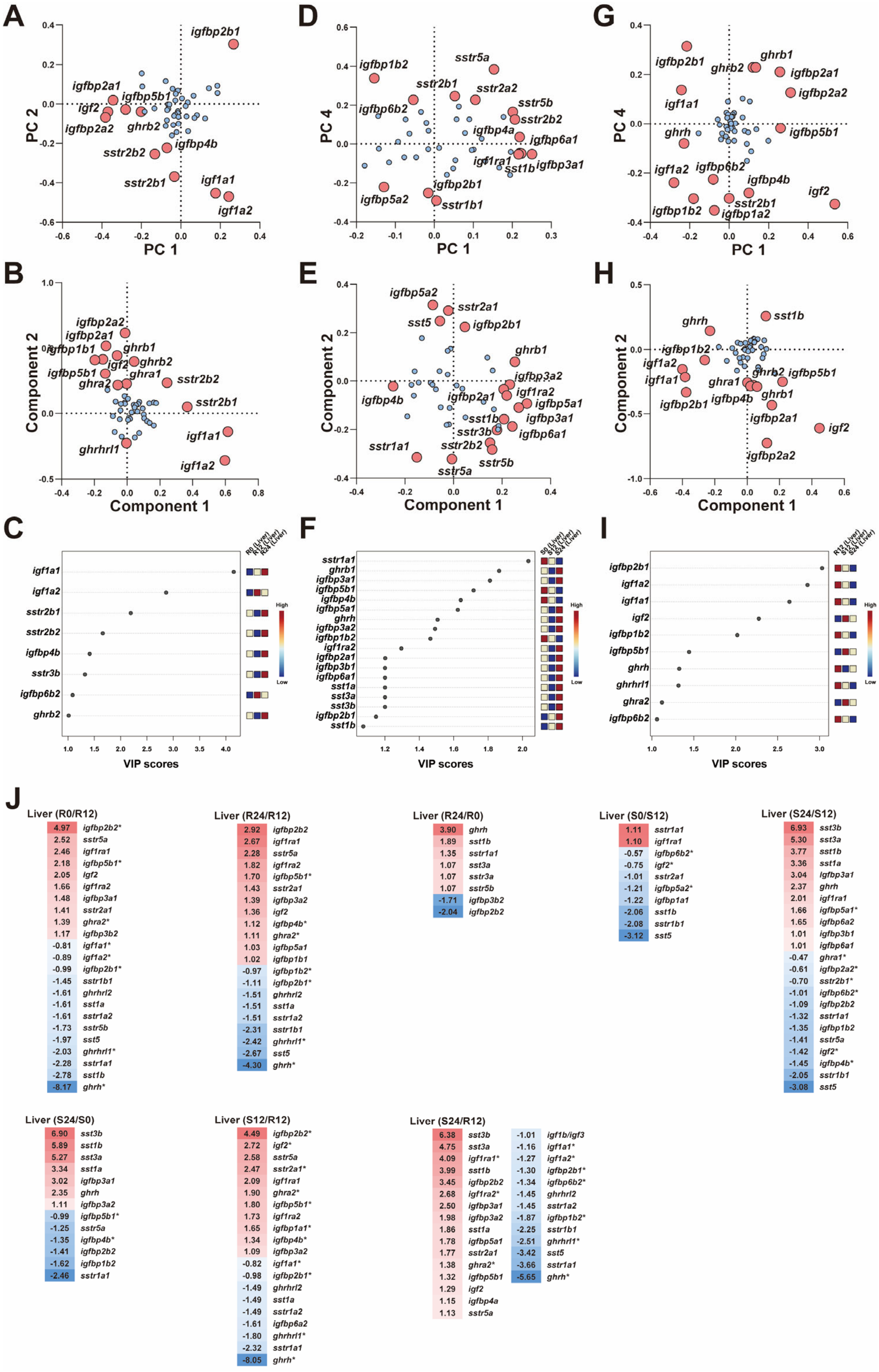

Figure S2. The key genes with changes in the liver GH-IGF axis in various environments. (**A-C**) PCA loading plot (**A**), PLS-DA loading plot (**B**), and variable importance in projection (VIP) values (**C**) of liver GH-IGF axis genes under different photoperiods in routine mode. (**D-F**) PCA loading plot (**D**), PLS-DA loading plot (**E**), and VIP values (**F**) of liver GH-IGF axis genes under different photoperiods in submerged mode. (**G-I**) PCA loading plot (**G**), PLS-DA loading plot (**H**), and VIP values (**I**) of liver GH-IGF axis genes in interactive environments of photoperiod and mode. (**J**) Differential expression between liver GH-IGF axis genes in the two groups. The numbers represent log2(fold change) values, and "*" indicates $P < 0.05$.

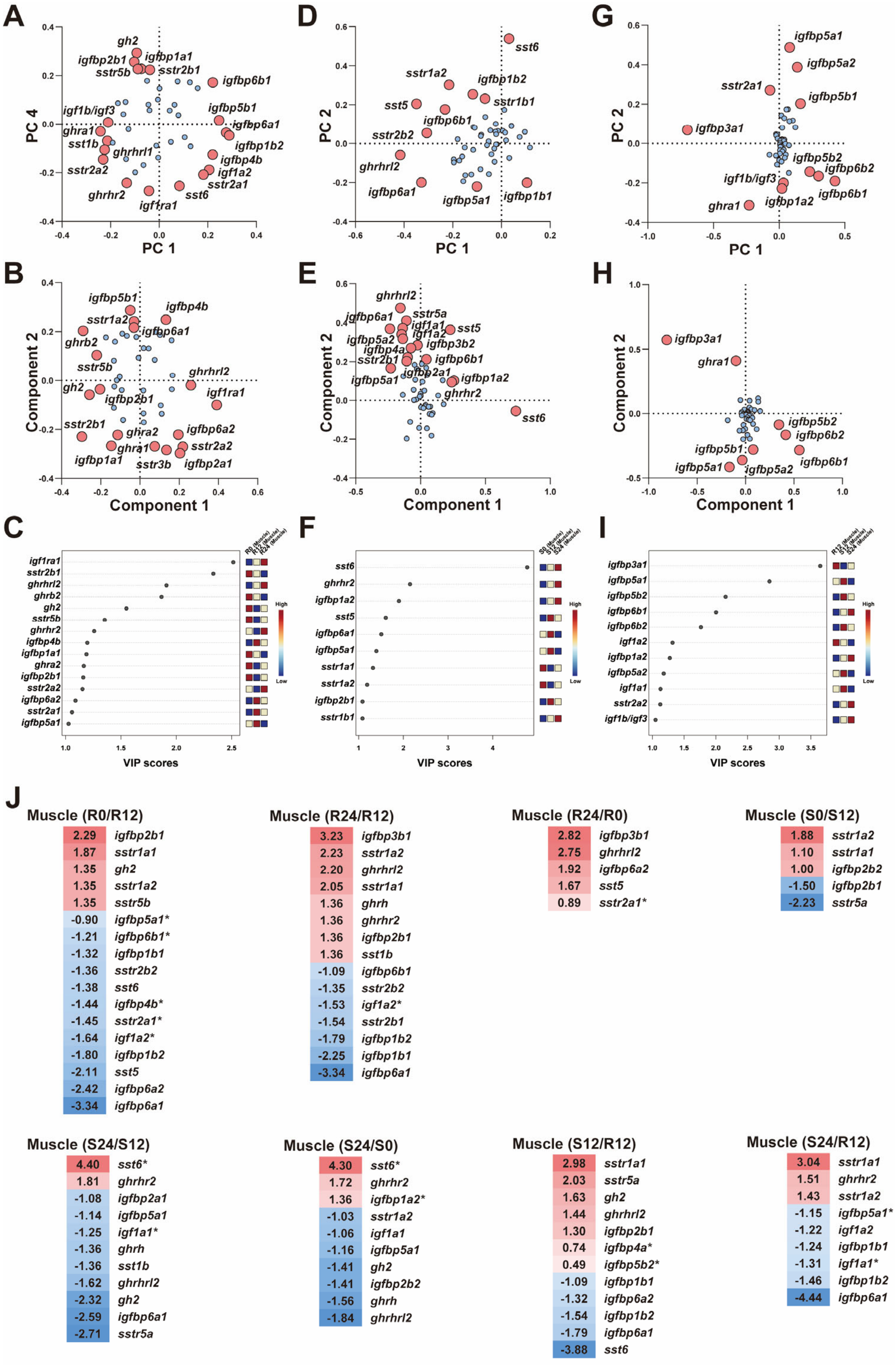

Figure S3. The key genes with changes in the muscle GH-IGF axis in various environments. (**A-C**) PCA loading plot (**A**), PLS-DA loading plot (**B**), and variable importance in projection (VIP) values (**C**) of muscle GH-IGF axis genes under different photoperiods in routine mode. (**D-F**) PCA loading plot (**D**), PLS-DA loading plot (**E**), and VIP values (**F**) of muscle GH-IGF axis genes under different photoperiods in submerged mode. (**G-I**) PCA loading plot (**G**), PLS-DA loading plot (**H**), and VIP values (**I**) of muscle GH-IGF axis genes in interactive environments of photoperiod and mode. (**J**) Differential expression between muscle GH-IGF axis genes in the two groups. The numbers represent log2(fold change) values, and "*" indicates $P < 0.05$.

Table S1. Sequence of primers used for qPCR validation.

Gene	Forward (5′-3′)	Reverse (5′-3′)
β-actin	GATGGGCCAGAAAGACAGCTA	TCGTCCCAGTTGGTGACGAT
sfxn1	GCACTTCTTCTTCGTCACTG	GGCTTCACCACTCCTAACC
che	CACCACTCCTCCACCAATC	TGTTCAGCGGCATTCCAA
ephexin-1	CACCAACCAGCCTCTCCT	CCATCATCCAGCCGTCATC
chrna3	ATGAGGAGCCGCAACAAG	TGACAGGAAGCCAATGACAG
bnc2	CCGACTACCACCTCTACCA	CCACCGCTACTGCTACTG
hba	CCAGACTAAGACCTACTTCTCC	ATCACTCCGACAGCATTACC
ifi44l	GCAGAAGCCAGCAGACTAA	CCTCCTCGTGGTAGTTCTTC
gbp	TCTTGGAGCAGTCTGTTACC	GCCACGCCATTGTTGTTG
mfap5	CTGCTCCTCTGCGGTTTC	GCCTCCTCTTCATTCCAGTC
mmp9	GCTCCTACCAGACCTTCCA	TGACATCACTCCACACCTTG
hsp70	ACCATCACCAACGACAAGG	CTCCACGCTGCTCTTCATAT
slap75	AGCGTGGCAGAGAAGAGA	CTCCTGTCCTCCGTCCTC
tdh	CACAACATCCTGGACATTGC	CGTAGCGGTGGTGGTAGT
myhc	CCAAGAAGGAAGCAGAACCA	CCGAAGCGAGACGAGTTG
lectin	GCGATGATGCTCCAGTGTA	CCAGCCTGTACCAGTCTTC
cuta	GCTGGCTGCTTGTGTCAA	GACCTCTGCTACTTCATACGG
gskbp	GATGCCTCCGAGAAGAAGTG	TGCTCCAGAACCTCCTCAG
arrdc2	TGTGGTGACGGATGAGGAG	GAGGTCGGAAGCGGAACT
dipp3-beta	CCGAACCAGACCAGAACTTA	TCACCAGCAACACCTCTTC

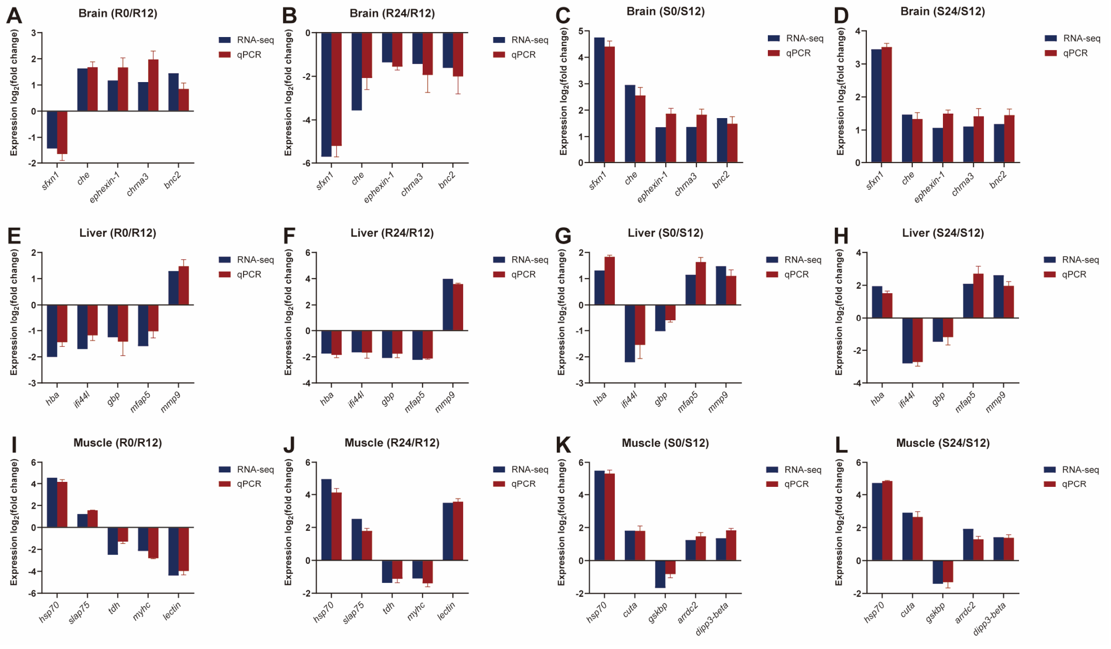

Figure S4. Validation of RNA-Seq results using qPCR. (A-D) The relative expression levels of differential genes in the brain in R0/R12 (A), R24/R12 (B), S0/S12 (C), and S24/S12 (D). (E-H) The relative expression levels of differential genes in the liver in R0/R12 (E), R24/R12 (F), S0/S12 (G), and S24/S12 (H). (I-L) The relative expression levels of differential genes in the muscle in R0/R12 (I), R24/R12 (J), S0/S12 (K), and S24/S12 (L).
